# Antioxidant Activity and Nutritional Status in Anorexia Nervosa: Effects of Weight Recovery

**DOI:** 10.3390/nu7042193

**Published:** 2015-03-30

**Authors:** María-Jesús Oliveras-López, Inmaculada Ruiz-Prieto, Patricia Bolaños-Ríos, Francisco De la Cerda, Franz Martín, Ignacio Jáuregui-Lobera

**Affiliations:** 1 Department of Molecular Biology and Biochemical Engineering, University of Pablo de Olavide of Seville, Ctra Utrera km 1, Seville 41013, Spain; E-Mails: mjolilop@upo.es (M.-J.O.-L.); fmarber@upo.es (F.M.); 2 Behavioral Sciences Institute, Seville 41011, Spain; E-Mails: inma.irp@gmail.com (I.R.-P.); pbr@tcasevilla.com (P.B.-R.); 3 DLCB Laboratory, Seville, 41010, Spain; E-Mail: fcodelacerdabar@terra.es; 4 CABIMER, Andalusian Center of Molecular Biology and Regenerative Medicine, University of Pablo de Olavide of Seville, Avda Americo Vespucio s/n, Seville 41092, Spain

**Keywords:** anorexia nervosa, nutritional status, diet therapy, catalase, total antioxidant capacity

## Abstract

Few studies are focused on the antioxidant status and its changes in anorexia nervosa (AN). Based on the hypothesis that renutrition improves that status, the aim was to determine the plasma antioxidant status and the antioxidant enzymes activity at the beginning of a personalized nutritional program (T_0_) and after recovering normal body mass index (BMI) (T_1_). The relationship between changes in BMI and biochemical parameters was determined. Nutritional intake, body composition, anthropometric, hematological and biochemical parameters were studied in 25 women with AN (19.20 ± 6.07 years). Plasma antioxidant capacity and antioxidant enzymes activity were measured. Mean time to recover normal weight was 4.1 ± 2.44 months. Energy, macronutrients and micronutrients intake improved. Catalase activity was significantly modified after dietary intake improvement and weight recovery (T_0_ = 25.04 ± 1.97 *vs.* T_1_ = 35.54 ± 2.60μmol/min/mL; *p* < 0.01). Total antioxidant capacity increased significantly after gaining weight (T_0_ = 1033.03 ± 34.38 *vs.* T_1_ = 1504.61 ± 99.73 μmol/L; *p* < 0.01). Superoxide dismutase activity decreased (*p* < 0.05) and glutathione peroxidase did not change. Our results support an association between nutrition improvement and weight gain in patients with AN, followed by an enhancement of antioxidant capacity and catalase antioxidant system.

## 1. Introduction

Anorexia nervosa (AN) is characterized by patient-induced and patient-maintained weight loss leading to progressive malnutrition, body image disturbance and fear of gaining weight. AN patients display nutritional and medical abnormalities including hypercholesterolemia [1,2,3].

AN patients usually consume less fat and more fiber than their healthy peers [4], although micronutrient deficits have also been observed in adult patients [5]. Restriction is greater during the more severe phases of the disorder [6]. Apart from the total calorie intake, other dietary parameters (e.g., diet energy density (DED) or diet variety (DV)) might predict a successful outcome in weight-recovered AN patients. In fact, the total calorie intake seems to be similar in patients with and without a successful outcome, but DED and DV are higher in the successful patient groups [7]. AN patients usually fall back on their initial eating patterns as treatment progresses, making it difficult to maintain any advances made in terms of increased calorie and macronutrient intake. In Spain, it has been reported that patients only reach 94% of the recommended intake in regard to the total energy content, although protein intake is maintained [8]. Finally, reduced calorie intake has been observed one year prior to illness onset and is especially evident as a reduction in fatty food intake [9].

AN may involve an insufficient intake of antioxidant vitamins and oligoelements [5,10] that are cofactors for the scavenging enzyme system, and glutathione [11]. AN causes chronic psychophysiological stress in the highly demanding period of adolescence [12]. Therefore, the generation of an excess of free radicals with increasing requirements of the scavenging system might combine with a decrease in the adaptive capacity to meet such demands [11].

AN patients have been compared with patients who display isolated protein-energy malnutrition (PEM) without AN [13]. Prior studies have evaluated antioxidant status; in marasmus cases, the pro-oxidant and antioxidant processes, which counteract each other, seem to decrease together [14]. Children with marasmus have increased pro-oxidant and decreased antioxidant status and the extent of oxidative stress increases with malnutrition severity [15]. Plasma antioxidant potential is reduced in marasmus due to an impaired antioxidant system, thus causing oxidant stress and peroxide formation [16]. The nutritional rehabilitation of children with different types of primary malnutrition has a positive impact on various antioxidant enzyme levels [17]. The catalase enzyme is one of the initial antioxidant defense mechanisms, followed by superoxide dismutase and gluthatione peroxidase. A higher antioxidant capacity has been correlated with health status and increased oxidative stress has been shown in illness [14].

However, there is only one study specifically focused on the antioxidant status among patients with AN [11], and there have been no follow-up studies addressing antioxidant status as result of the re-nutrition.

The aim of this study was to determine the plasma antioxidant status and catalase, superoxide dismutase and gluthatione peroxidase activity in AN at the beginning of the treatment as well as when patients have recovered a normal body mass index (BMI ≥ 18.5 kg/m^2^) and maintained it for at least one month. In addition, the relationship between the change in antioxidant status and biochemical parameters was determined.

## 2. Materials and Methods

### 2.1. Study Participants

Twenty-five women, aged 19.20 ± 6.07 years, diagnosed with AN (restrictive subtype) according to the Diagnostic and Statistical Manual of Mental Disorders, fourth edition, text revision (DSM-IV-TR) [18] criteria participated in the study. Their initial BMI was 16.80 ± 1.04, the mean duration of illness was 6.40 ± 3.12 months, and they were receiving treatment as outpatients in the Eating Disorders Unit of the Behavioral Sciences Institute (EDUBSI). Subjects with associated comorbid physical pathologies as well as other DSM IV-TR Axis I disorders, were excluded from the study. None of the subjects were taking any medications known to affect nutritional status or regulation of fat metabolism, and they were not taking any supplements. They were not allowed to practice physical exercise. The patients’ parents were clearly instructed to supervise their physical activity. Ethical approval for the study was obtained from the corresponding committee of the Behavioral Sciences Institute, and written informed consent from each subject (and from the parents when appropriate) enrolled into the study was obtained. All procedures complied with the Declaration of Helsinki. Although 48 patients were initially recruited for the study, only 25 fit the final inclusion criteria and were able to undergo the study.

### 2.2. Study Design and Procedure

The patients were studied on two occasions, in association with the therapeutic schedule that was established where they were treated. The first session (T_0_) took place within the first week after starting treatment and the second session (T_1_) after the subjects reached a BMI ≥ 18.5 (according to other authors’ criteria) [19,20], and being sure that weight was maintained at least one month before the second blood test.

The study lasted 36 months. After an initial evaluation (by means of a clinical interview), anthropometric (weight, height, BMI) and body composition measurements, analyses of the nutritional intake and blood samples were obtained. All patients attended the EDUBSI twice a week. The personalized nutritional program was performed according to a previous paper [21], and a qualified nutritionist led the patients’ nutritional rehabilitation by means of that program. Considering standard practices for measuring food intake in these patients [4,22,23], the study was designed to use 24 h dietary recalls as well as digital photography (before and after meals) to determine the adherence to the diet. Both measurement types (recalls and photographs) were assigned to the patients’ parents in order to improve the reliability of this information.

During the T_0_ well-trained nutritionists requested patients (with parental supervision) to complete the 24 h dietary recall and to complete this task by means of food photographs without starting feeding rehabilitation since this previous intake assessment was part of the initial dietary survey. Afterwards, the treatment started with the nutritional rehabilitation process. The initial energy recommendation was based on low-energy diets (800–1500 kcal/day), which was progressively increased up to 2500–3000 Kcal/day, with a weekly weight gain expected to be<1 kg [21]. Because there is no *gold standard* method in the energy requirement’s determination in anorexia nervosa patients, the energy increases was carried out using the empirical method, which is considered the most appropriate method in clinical practice [24].

Recovery was evaluated only at the physical level, which in this case implied a weight criterion (we did not consider other recovery criteria for this study) of a BMI ≥18.50, the cut-off point for the normal range in the international classification system for underweight, overweight and obesity (World Health Organization, WHO) [25]. In the case of patients under 19 years, a BMI ≥ 18.50 is also internationally accepted as normal (WHO). In addition, a BMI at least of 18.5 clearly indicates weight restoration, especially for these types of patients [19,20]. All measurements were performed at T_0_ and T_1_.

### 2.3. Nutritional Intake

The program DIAL 1.0 (Alce Ingeniería, Madrid, Spain) [26] was used to assess calorie content, macronutrients and the proportion of calories derived from them, micronutrients and trace elements.

### 2.4. Anthropometric and Body Mass Index Measurements

BMI was calculated according to standard procedures (weight-kg-/height-m^2^). Height was measured using a wall-mounted stadiometer (Holtain, Dyfed, UK), to the nearest 0.5 cm, with the participant’s head in the Frankfort plane. Weight and body composition were assessed by tetrapolar bioelectrical impedance foot-foot (50 kHz—measurement frequency, 500 μA—measurement current, 0.1 kg and 0.1% precision) by means of a Tanita monitor BC 420 MA (TANITA Europe GmbH, Sindelfingen, Germany) to the nearest 0.1 kg. Measurements were performed with the participants wearing light indoor clothing and neither shoes nor metallic fittings. A correction factor of −1 kg was used to adjust for the weight of clothes. In order to minimize the well-known error when analyzing body composition by bioelectrical impedance, standardized protocol were applied measuring the weight and body composition barefoot in supine position and inferior limbs at 45° abduction. Participants were encouraged to fast at least two hours before the test, avoiding alcohol, coffee, soft drinks with caffeine and chocolate during the past 24 h. In addition, they were advised to have urinated half an hour before and not having made strenuous physical exercise in the past 24 h. Each measure was assessed at the same time, neither on premenstrual nor menstrual period. The bioelectrical impedance equipment was on a non-conductive surface and in a not extreme room temperature [27,28].

### 2.5. Blood Collection and Processing for Biochemical and other Determinations

Blood samples were taken from 08.30 to 09.00 in the morning after a 12 h fast at the first session (T_0_) and at the second session (T_1_). Blood was obtained by antecubital venipuncture of the non-dominant forearm. Blood for the hematology and biochemical determination was collected in a vacutainer tube with lithium heparin (BD Vacutainer, Becton Dickinson, Madrid, Spain). Hemogram and biochemical parameters were measured by means of the Abbott Cell Dyn 3500 (Santa Clara, CA, USA) automated hematology analyzer and the automatic biochemistry analyzer SPINTECH 240 (Girona, Spain), respectively. For antioxidant enzymes analysis, blood was collected in a tube with sodium citrate (BD Vacutainer, Madrid, Spain). The samples were stored in the dark in containers with ice and processed within one hour after extraction. Plasma was separated by centrifugation of the blood samples at 1500 rpm for 20 min at 18–25 °C. All plasma samples were aliquoted for total antioxidant capacity and enzymes measurements in cryovials and stored at −80 °C until all analyses were performed.

### 2.6. Biochemical Parameters Measurements

Glucose, urea, creatinine, albumin, prealbumin, total protein, total cholesterol, high-density lipoprotein (HDL), low-density lipoprotein (LDL) and triglycerides were determined. The biochemical parameters were determined by means of colorimetric and enzymatic tests, both measured by spectrophotometry.

### 2.7. Total Antioxidant Capacity Measurements

Antioxidant capacity was measured by spectrophotometric assays using the PAO assay kit (OXIS Research, Inc., Madrid, Spain) in plasma samples. The total antioxidant capacity (TAC) assay was based on the reduction of copper using bathocuproine sulfonate as a cuprous-chelating ligand. The assay results are expressed as Uric Acid equivalents in μmol/L unit, and all analytical determinations were run in triplicate.

### 2.8. Antioxidant Enzymes Activity Measurements

Antioxidant activity of plasma catalase (CAT), superoxide dismutase (SOD) and gluthatione peroxidase (GPX) were measured by spectrophotometric assays using commercial kits, according to the manufacturer’s instructions (Cayman Chemicals, Paris, France). This method assesses the functional capacity of the enzyme to act on its substrate. CAT activity was measured using the peroxidative function of CAT. Total SOD activity was measured using a tetrazolium salt for detection of superoxide radicals generated by xanthine oxidase and hypoxanthine. GPX activity was indirectly determined by a coupled reaction with glutathione reductase. The activities are given in μmol/min/mL, and all determinations were run in triplicate.

### 2.9. Data Analysis

Data were analyzed with the statistical package, SPSS 17.0. (SPSS, Inc., Chicago, IL, USA, 2007). The significance level was established at *p*< 0.05. Values are expressed as the means and standard deviations (X ± SD), except enzyme activity data and antioxidant capacity, which are expressed as the means with standard errors of the mean (X ± SEM). The variable “magnitude of change” (T_0_–T_1_) was calculated for each variable and the normality of these transformed variables was checked by the Kolmogorov–Smirnov test. As a result, the parametric Student’s *t*-test was applied (despite a sample size lower than 30, a *t*-test is considered to be appropriate when the variables fit the normal distribution). In the case of correlations, Pearson’s r coefficient was calculated.

## 3. Results

### 3.1. AN Patients’ Characteristics

The mean time to recovery (BMI ≥ 18.50) was 4.1 ± 2.44 months. Patients’ BMI and body composition (at T_0_ and T_1_) are summarized in Table 1. All changes were statistically significant.

**Table 1:** Body mass index and body composition (mean and SD) at Time 0 and Time 1.

	Time 0	Time 1
**Body Mass Index (kg/m^2^)**	16.80 ± 1.04	19.43 ± 0.70 *
**Fat mass (%)**	10.24 ± 4.95	16.80± 3.78 **
**Lean mass (%)**	89.76 ± 4.95	83.19 ± 3.77 **
**Total body water (%)**	65.59 ± 3.83	60.83 ± 2.88 **

* *p* < 0.05; ** *p* < 0.01.

### 3.2. Hematological and Biochemical Parameters

All of the plasma hematological and biochemical parameters measured at T_0_ and T_1_ are shown in Table 2.

**Table 2:** Hematological and biochemical parameters at Time 0 and Time 1.

	Time 0	Time 1
**Hematological Parameters**		
Erythrocytes (million/mm^3^)	4.38 ± 0.50	4.51 ± 0.52*
Hemoglobin (g/100 mL)	13.29 ± 1.32	13.53 ± 1.17
Hematocrit (mL/100 mL)	40.33 ± 4.17	41.32 ± 3.78*
Mean Corpuscular Volume (mm^3^)	92.33 ± 3.48	92.02 ± 4.83
Mean Corpuscular Hemoglobin (pg/cell)	30.46 ± 1.73	30.18 ± 2.27
Mean Corpuscular Hemoglobin Concentration (g/100 mL)	32.99 ± 1.28	32.78 ± 1.36
Leucocytes/mm^3^	6.14 ± 1.53	6.33 ± 1.57
Lymphocytes (%)	40.84 ± 8.21	35.25 ± 7.66 **
**Biochemical Parameters**		
Glucose (mg/dL)	81.20 ± 8.63	84.08 ± 11.42
Urea (mg/dL)	32.61 ± 8.20	32.91 ± 7.74
Creatinine (mg/dL)	0.71 ± 0.07	0.66 ± 0.07 *
Albumin (g/dL)	4.59 ± 0.22	4.65 ± 0.25
Prealbumin (mg/dL)	30.08 ± 2.90	30.21 ±3.38
Total proteins (g/100 dL)	7.10 ± 0.57	7.01 ± 0.60
Cholesterol (mg/dL)	184.20 ± 33.83	179.88 ± 39.52
HDL-Cholesterol (mg/dL)	60.08 ± 18.00	68.08 ± 18.31 *
LDL-Cholesterol (mg/dL)	112.04 ± 36.46	98.98 ± 31.12 *
Triglycerides (mg/dL)	77.80 ± 39.47	66.04 ± 17.89 *

* *p* < 0.05; ** *p* < 0.01.

### 3.3. Dietary Characteristics

The calorie content of the diet and the macronutrient intake (at T_0_ and T_1_) are shown in Table 3, along with the statistical significance. The micronutrients most related to antioxidant defense status are also shown.

**Table 3:** Nutrient intake at Time 0 and Time 1 (macronutrients and vitamins).

	Time 0	Time 1
**Energy (Kcal/day)**	1341.64 ± 621.36	1880.60 ± 454.62 **
**Macronutrients (day)**		
Proteins (g)	52.57 ± 21.63	104.10 ± 18.95 **
Carbohydrates (g)	135.25 ± 69.00	291.68 ± 57.15 **
Total fat (g)	62.41 ± 30.19	137.80 ± 22.81 **
**Micronutrients (day)**		
Vitamin B_1_ (mg)	0.93 ± 0.47	1.83 ± 0.38 **
Vitamin B_2_ (mg)	1.31 ± 0.58	2.29 ± 0.36 **
Vitamin B_3_-Niacin (mg)	22.32 ± 9.61	45.43 ± 8.62 **
Vitamin B_9_-Folic acid (μg)	196.09 ± 101.76	397.92 ± 71.69 **
Vitamin C (mg)	111.37 ± 62.30	194.48 ± 49.51 **
Vitamin A (μg retinol equivalents)	601.08 ± 307.55	1268.20 ± 246.22 **
Retinol (μg)	260.69 ± 146.38	564.00 ± 123.38 **
Beta-carotene (μg)	1746 ± 960.12	3840 ± 1211.38 **
Vitamin E (μg alpha-tocopherol equivalents)	6.27 ± 3.42	11.82 ± 2.90 **
Iron (mg)	8.89 ± 4.44	18.08 ±3.26 **
Magnesium (mg)	180.02 ± 80.73	369.20 ± 61.74 **
Zinc (mg)	5.77 ± 2.42	10.80 ± 1.96 **
Selenium (μg)	67.67 ± 28.95	211.40 ± 29.05 **
Copper (mg)	0.79 ± 0.37	1.71 ± 0.29 **
Manganese (mg)	1.72 ± 1.30	3.34 ± 0.58 **
Beta-sisosterol (mg)	16.98 ± 13.07	37.39 ± 11.85 **
Campesterol (mg)	1.52 ± 1.34	4.05 ± 1.46 **
Stigmasterol (mg) 3.23 ± 2.55		7.94 ± 3.29 **

** *p* < 0.01.

### 3.4. Plasma Antioxidant Enzymes Activity

**Figure 1:**
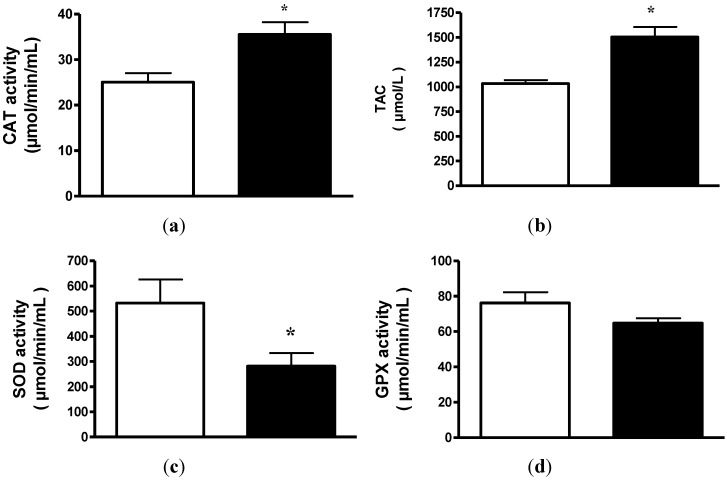
(**a**) Plasma catalase (CAT) activity from anorexia nervosa (AN) patients before (white) and after (black) weight gain. The results are reported as the means ± SEM (*n* = 25),* *p* < 0.01; (**b**) Plasma total antioxidant capacity (TAC) from anorexia nervosa (AN) patients before (white) and after (black) weight gain. The results are reported as the means ± SEM (*n* = 25),* *p* < 0.01; (**c**) Plasma superoxide dismutase (SOD) activity from anorexia nervosa (AN) patients before (white) and after (black) weight gain. The results are reported as the means ± SEM (*n* = 25),*p < 0.05; (**d**) Plasma glutathione peroxidase (GPX) activity from anorexia nervosa (AN) patients before (white) and after (black) weight gain. The results are reported as the means ± SEM (*n* = 25).

CAT activity significantly increased in patients after dietary intake improvement and consequent weight recovery (T_0_ = 25.04 ± 1.97 *vs.* T_1_ = 35.54 ± 2.60 μmol/min/mL; *p* < 0.01). In 21 out of 25 cases, CAT increased within the period of time of the study (Figure 1a). However, SOD activity (Figure 1c) significantly decreased after weight recovery in all patients (T_0_ = 532.5 ± 93.4 *vs.* T_1_ = 281.4 ± 52.03 μmol/min/mL; *p* < 0.05) and GPX (Figure 1d) did not show significant differences (T_0_ = 76.2 ± 6.08 *vs.* T_1_ = 64.8 ± 2.7 μmol/min/mL; n.s.).

### 3.5. Total Antioxidant Capacity

TAC increased significantly after weight gain (T_0_ = 1033.03 ± 34.38 *vs.* T_1_ = 1504.61 ± 99.73 μmol/L; *p* < 0.01). In this case, an increase in TAC was observed in 22 patients (Figure 1b).

### 3.6. Distribution of the Sample with Respect to the Change in TAC/CAT

**Figure 2:**
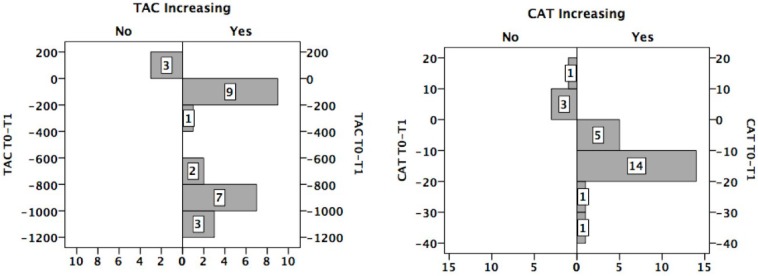
Number of patients who increased (Yes) or not (No) their total antioxidant capacity/catalase (TAC/CAT).

Despite an improvement in dietary intake and BMI, TAC and CAT decreased within the period of treatment in three and four patients, respectively (Figure 2).

### 3.7. TAC/CAT Activity and Mineral Ingestion

Comparing patients with and without TAC improvement, the increase in iron intake was significantly greater in the first group than in the latter one (9.98 mg *vs.* 3.53 mg; *p* < 0.01). No significant differences with respect to selenium, copper, magnesium, zinc and manganese were found.

Comparing patients with and without CAT improvement, the increase in the following minerals was greater in the first group: selenium (80.19 μg *vs.* 50.10 μg; *p* < 0.05), copper (0.95 mg *vs.* 0.69 mg; *p* < 0.05), magnesium (203.03 mg *vs.* 116.42; *p* < 0.01), zinc (5.33 mg *vs.* 3.42 mg; *p* < 0.05), manganese (1.95 *vs.* 0.16 mg; *p* < 0.01) and iron (9.95 mg *vs.* 5.32 mg; *p* < 0.05).

### 3.8. Correlations between the Magnitude of Change in TAC/CAT and the Experimental Variables

**Figure 3:**
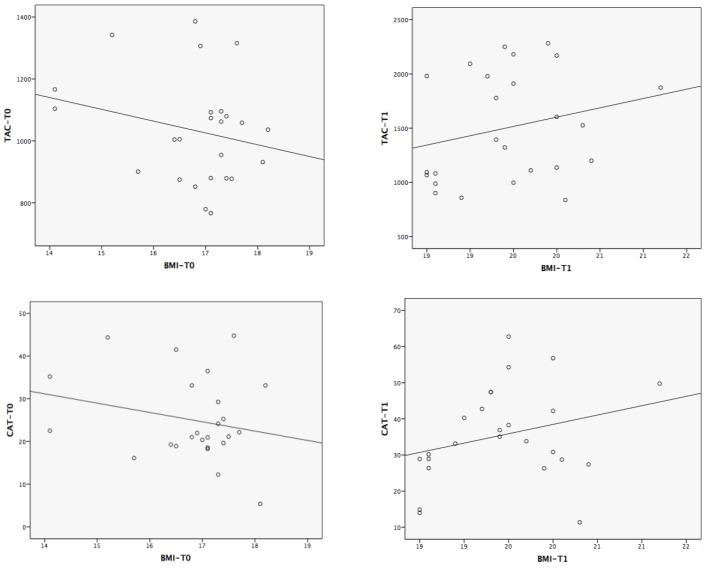
Correlations between body mass index (BMI) and total antioxidant capacity/catalase (TAC/CAT) at T_0_ and T_1_.

Potential associations between the change in anthropometric, hematologic, biochemical and nutritional variables and the change in TAC/CAT were analyzed. Despite not being statistically significant, after weight recovery, a change in the tendency was observed with respect to the association (Pearson’s r) between the changes in BMI and TAC/CAT (from −0.23 to 0.10 for TAC and from −0.23 to 0.24 for CAT) (Figure 3).The correlations between the change in TAC/CAT and the changes in the main antioxidant status-related variables are shown in Table 4.

**Table 4:** Correlations between change in catalase (CAT) and total antioxidant capacity (TAC) and the magnitude of change in antioxidant function-related variables.

	CAT-Difference (T_0_ − T_1_)	TAC-Difference (T_0_ − T_1_)
**Body Mass Index**	−0.48 *	−0.25
**Nutrients Intake**		
Energy	−0.72 **	−0.15
Proteins	−0.60 **	−0.21
Vitamin B_1_	−0.41	−0.04
Vitamin B_2_	−0.35	−0.04
Vitamin B_3_	−0.51 *	−0.28
Vitamin B_9_	−0.45 *	−0.11
Vitamin C	−0.12	−0.10
Vitamin A	−0.40	−0.36
Retinol	−0.35	−0.05
Beta-Carotene	−0.31	−0.39
Vitamin E	−0.29	−0.41
Iron	−0.53 *	−0.21
Magnesium	−0.60 **	−0.25
Zinc	−0.54 *	−0.24
Selenium	−0.35	−0.39
Copper	−0.76 **	−0.33
Manganese	−0.59 **	−0.13
Sisosterol	−0.38	−0.33
Campesterol	−0.45 *	−0.14
Stigmasterol	−0.33	−0.13

* *p* < 0.05; ** *p* < 0.01.

## 4. Discussion

AN patients show disordered nutrient intakes that may persist despite outpatient interventions; therefore, it is important to gather information that may improve strategies for nutritional counseling and weight rehabilitation [4]. For instance, significant micronutrient deficiencies among patients with AN have been observed [5].

In this study, patients’ recovery was focused on nutritional rehabilitation and weight restoration, which are the main objectives in the early treatment of AN patients [29,30]; these recovery parameters were likely to most closely relate to changes in antioxidant activity. The mean time to recovery (BMI ≥ 18.5) was 4.1 ± 2.44 months, which is normal considering that patients in similar programs usually regain near normal weight within six months [30,31].

An imbalance between oxidative stress and antioxidant capacity results in several diseases [32,33] and an inadequate diet contributes to significantly increased production of reactive oxygen species (ROS) [34,35]. Prior studies have shown that TAC serves as a sufficient indicator of plasma individual antioxidant status and explains the overall diet quality as well as the endogenous antioxidant defense status [36,37].

PEM, including AN, is associated with a depleted antioxidant status and subsequent susceptibility to oxidative stress and damage [38,39]. CAT activity decreases in several diseases [40,41], and animal studies show that dietary interventions, such as high-protein diets, can modify CAT levels [42]. In a previous paper, antioxidant status in AN was studied by means of vitamin E and antioxidant enzymes in patients with AN. Diminished vitamin E levels and increased catalase activity in erythrocytes of malnourished anorexic patients [11] has also been shown, probably as an adaptive initial mechanism of the enzyme.

Certain minerals are involved in enzyme activities. Low levels of zinc and selenium have been related to lower plasma CAT activity in asthmatic patients [43]. We found that patients who showed increased CAT activity also showed higher intake of selenium, copper, magnesium, zinc, manganese and iron. Despite not finding significant changes in CAT activity, previous researchers found that dietary iron restriction diminishes the production of free radicals via Fenton and Haber-Weiss reactions in anemic rats [44]. Our results show a negative correlation between the magnitude of change with respect to iron intake and CAT activity, so that larger changes in iron consumption produced smaller changes in CAT activity. An explanation could be a compensatory mechanism of the enzyme against ROS production.

Correlations between the magnitudes of change of CAT and nutrients intake (Table 4) showed that higher increases in CAT activity correlated negatively with higher increases in nutrient ingestion. We hypothesize that there is a limited amount of nutrients over which higher levels of intake do not influence additional increases in CAT levels. This enzyme is one of the initial responses against oxidative stress, and its progressive normalization through re-feeding may decrease its role in favor of other systems.

The serum TAC levels, measured by means of ORAC, have been shown to decrease by 24% for AN with respect to healthy controls [45]. Similarly, the level of oxidative stress was significantly lower in marasmus cases compared with controls [14] and children with PEM are potentially susceptible to high oxidative stress [46]. Our patients showed decreased antioxidant status, improved after re-nutrition. These results are also consistent with the work of others authors that found better antioxidant status after re-nutrition by a different mechanism [17]. Considering pro-oxidant and antioxidant status, marasmic children have increased pro-oxidant and decreased antioxidant status [17], the latter associated with significant peroxide production [16]. This decreased antioxidant status has been reported to be improved after nutritional treatment [17].

The studies support that TAC serves as a sufficient indicator of plasma individual antioxidants that were found to be significant and negative (especially energy, proteins, magnesium, copper and manganese). These significant correlations were found for CAT but not TAC levels.

Some relevant questions arise considering the fact that not all patients showed a change in the same direction (in fact, seven patients showed a decrease either in TAC or CAT levels). Is there a time limit for the duration of weight loss, beyond which the normalization of antioxidant status would be more difficult? Would a longer period of normal eating and weight maintenance be required to improve the antioxidant status?

To our knowledge, there are no studies with humans concerning the relationship between nutritional status after treatment and oxidative stress. After re-nutrition and normalization of the BMI, patients improved their TAC levels and CAT activity. This suggests that plasma antioxidant capacity could be a good predictor of nutritional status in these patients. An explanation could be that an adequate diet and the consequent improvement of the nutritional status could cause a suitable cellular redox status, so that CAT would not be wasted and would work properly to eliminate hydrogen peroxide. CAT is important when hydrogen peroxide is present at high concentrations and diseases of malnutrition have been shown to produce high levels of ROS. Therefore, our results suggest that diet improvement and weight recovery improve CAT activity, leading to a reduction in the ROS levels formed during AN. The adequacy of the diet and the enhanced activity of CAT seem to contribute to better TAC levels after weight recovery. Thus, re-nutrition induces recuperation of the antioxidant defensive system, which is able to respond to the increased free radical production observed in AN.

After the renutrition program and the consequent normalization of BMI, patients reduced SOD activity and did not modified GPX. The combined defensive activity of both SOD and GPX with CAT would explain these results. The higher CAT activity after renutrition would reduce the circulating hydrogen peroxide level thus inducing a reduction in SOD activity (adaptive response). The reduction of SOD activity may be due to low levels of superoxide radical anion. In addition, GPX would not be modified because it is not necessary to eliminate hydrogen peroxide, which was previously removed by CAT. All these antioxidant mechanisms are associated to the higher plasma TAC levels found.

Some studies with enzymes usually reveal that SOD and GS-PX activities increase in several diseases and oxidative stress states because more antioxidant requirements are needed [40,47,48,49]. The only research related to AN [11] did not study changes with re-nutrition. They found an increased in CAT, a decreased in SOD and the same activity for GPX between controls and patients. In a previous paper with marasmic children [17], authors found changes in SOD and GPX activities before and after nutrition rehabilitation. In this study, both were significantly lower in malnourished and significantly increased after nutritional rehabilitation. However, these authors did not study changes in CAT activity, thus not being clear the defensive pathway.

The main limitation of our research was that at T_1_, the BMI threshold (≥18.5) is at the lower limit of the normal weight range. However, this is the cut-off point used to evaluate the normal range in the international classification system, and it clearly indicates weight restoration, especially for these patients. The lack of biochemical measurements in tissues is another limitation of the study, although it has been widely confirmed that an increase in both antioxidant markers in plasma correlates with improvement of the whole-body antioxidant defense system.

## 5. Conclusions

Our results show a positive association between dietary intake improvement and antioxidant status, measured by TAC levels and CAT activity and they support a positive association between improvements in nutrition and the enhancement of antioxidant capacity and the catalase antioxidant system in AN patients.

## References

[1] Muñoz M.Y., Argente J. (2002). Anorexia nervosa in femaleadolescents: Endocrine and bone mineral densitydisturbances. Eur. J. Endocr..

[2] Feillet F., Feillet-Coudray C., Bard J.M., Parra H.J., Favre E., Kabuth B., Fruchart J.C., Vidailhet M. (2000). Plasma cholesterol and endogenous cholesterol synthesis during refeeding in anorexia nervosa. Clin. Chim. Acta.

[3] Jáuregui-Garrido B., Bolaños-Ríos P., Santiago-Fernández M.J., Jaúregui-Lobera I. (2012). Lipid profile and cardiovascular risk in anorexia nervosa; the effect of nutritional treatment. Nutr. Hosp..

[4] Misra M., Tsai P., Anderson E.J., Hubbard J.L., Gallagher K., Soyka L.A., Miller K.K., Herzog D.B., Klibanski A. (2006). Nutrient intake in community-dwelling adolescent girls with anorexia nervosa and in healthy adolescents. Am. J. Clin. Nutr..

[5] Hadigan C., Anderson E., Miller K., Hubbard J., Herzog D., Klibanski A., Grinspoon S.K. (2000). Assessment of macronutrient and micronutrient intake in women with anorexia nervosa. Int. J. Eat. Disord..

[6] Beaumont P., Chambers T., Rouse L., Abraham S. (1981). The diet composition and nutritional knowledge of patients with anorexia nervosa. J. Hum. Nutr..

[7] Schebendach J.E., Mayer L.E., Devlin M.J., Attia E., Contento I.R., Wolf R.L., Walsh B.T. (2008). Dietary energy density and diet variety as predictors of outcome in anorexia nervosa. Am. J. Clin. Nutr..

[8] Nova E., Varela P., López-Vidriero I., Toro O., Ceñal M.J., Casas J., Marcos A. (2001). A one-year follow-up study in anorexia nervosa. Dietary pattern and anthropometrical evolution. Eur. J. Clin. Nutr..

[9] Affenito S.G., Dohm F.A., Crawford P.B., Daniels S.R., Striegel-Moore R.H. (2002). Macronutrient intake in anorexia nervosa: The National Heart, Lung, and Blood Institute Growth and Health Study. J. Pediatr..

[10] Winston A.P. (2012). The clinical biochemistry of anorexia nervosa. Ann. Clin. Biochem..

[11] Moyano D., Sierra C., Brandi N., Artuch R., Mira A., García-Tornel S., Vilaseca M.A. (1999). Antioxidant status in anorexia nervosa. Int. J. Eat. Disord..

[12] Jáuregui-Lobera I., Ezquerra-Cabrera M., Carbonero-Carreño R., Ruiz-Prieto I. (2013). Weight misperception, self-reported physical fitness, dieting and some psychological variables as risk factors for eating disorders. Nutrients.

[13] Nova E., Samartin S., Gomez S., Morande G., Marcos A. (2002). The adaptive response of the immune system to the particular malnutrition of eating disorders. Eur. J. Clin. Nutr..

[14] Celik M., Sermatov K., Abuhandan M., Zeyrek D., Kocyigit A., Iscan A. (2012). Oxidative status and DNA damage in children with marasmic malnutrition. J. Clin. Lab. Anal..

[15] Ece A., Gürkan F., Celik F., Boşnak M., Yel S., Balik H., Erel O. (2007). Paraoxonase, total antioxidant activity and peroxide levels in marasmic children: Relationships with leptin. Clin. Biochem..

[16] Catal F., Avci A., Karadag A., Alioglu B., Avci Z. (2007). Oxidant and antioxidant status of Turkish marasmic children: A single center study. J. Trace Elem. Med. Biol..

[17] Shaaban S.Y., Nassar M.F., Ibrahim S.A., Mahmoud S.E. (2002). Impact of nutritional rehabilitation on enzymatic antioxidant levels in protein energy malnutrition. East Mediterr. Health J..

[18] (2000). Diagnostic and Statistical Manual of Mental Disorders: DSM IV-RT.

[19] Scalfi L., Potito A., Bianchi L., Marra M., Caldara A., Nicolai E., Contaldo F. (2002). Body composition changes in patients with anorexia nervosa after complete weight recovery. Eur. J. Clin. Nutr..

[20] Meguerditchian C., Samuelian-Massat C., Valéro R., Begu-Le Corroller A., Fromont I., Mancini J., Sparrow J.D., Poinso F., Vialettes B. (2009). The impact of weight normalization on quality of recovery in anorexia nervosa. J. Am. Coll. Nutr..

[21] Jáuregui-Lobera I., Bolaños P. (2012). Revisión del tratamientodietético-nutricional de la anorexia nerviosa. Rev. Med. Chile.

[22] Schebendach J.E., Mayer L.E., Devlin M.J., Attia E., Contento I.R., Wolf R.L., Walsh T. (2011). Food choice and diet variety in weight-restored patients with anorexia nervosa. J. Am. Diet. Assoc..

[23] Schebendach J., Mayer L.E., Devlin M.J., Attia E., Walsh B.T. (2012). Dietary energy density and diet variety as risk factors for relapse in anorexia nervosa: A replication. Int. J. Eat. Disord..

[24] Birmingham C.L., Hlynsky J., Whiteside L., Geller J. (2005). Caloric requirement for refeedinginpatients with anorexia nervosa: The contribution of anxiety exercise, and cigarette smoking. J. Eat. Disord..

[25] World Health Organisation (WHO) (2000). Obesity: Preventing and Managing the Global Epidemic.

[26] (2008). Programa para la evaluación de dietas y gestión de datos de alimentación.

[27] Kyle U.G., Bosaeus I., de Lorenzo A.D., Deurenberg P., Elia M., Gómez J.M., Heitmann B.L., Kent-Smith L., Melchior J.C., Pirlich M. (2004). Composition of the ESPEN Working Group. Bioelectrical impedance analysis—Part I: Review of principles and methods. Clin.Nutr..

[28] Kyle U.G., Bosaeus I., de Lorenzo A.D., Deurenberg P., Elia M., Gómez J.M., Heitmann B.L., Kent-Smith L., Melchior J.C., Pirlich M., Scharfetter H. (2004). Bioelectrical impedance analysis—Part II: Utilization in clinical practice. Clin. Nutr..

[29] Golden N.H., Meyer W. (2004). Nutritional rehabilitation of anorexia nervosa. Goals and dangers. Int. J. Adolesc. Med. Health.

[30] El Ghoch M., Calugi S., Lamburghini S., Dalle Grave R. (2014). Anorexia nervosa and body fat distribution: A systematic review. Nutrients.

[31] Lozano-Serra E., Andrés-Perpiña S., Lázaro-García L., Castro-Fornieles J. (2014). Adolescent anorexia nervosa: Cognitive performance after weight recovery. J. Psychosom. Res..

[32] Cumurcu B.E., Ozyurt H., Etikan I., Demir S., Karlidag R. (2009). Total antioxidant capacity and total oxidant status in patients with major depression: Impact of antidepressant treatment. Psychiatry Clin. Neurosci..

[33] Kolling J., Scherer E.B., da Cunha A.A., da Cunha M.J., Wyse A.T. (2011). Homocysteine induces oxidative-nitrative stress in heart of rats: Prevention by folic acid. Cardiovasc. Toxicol..

[34] Dandana A., Ferchichi S., Addad F., Jaidane Z., Chahed H., Gammoudi I., Chalghoum A., Bricca G., Miled A. (2011). Evaluation of oxidative stress among coronary diabetics patients. Acta Biomed..

[35] Kim Y.J., Ahn Y.H., Lim Y., Kim J.Y., Kim J., Kwon O. (2013). Daily nutritional dose supplementation with antioxidant nutrients and phytochemicals improves DNA and LDLstability: A double-blind, randomized, and placebo-controlled trial. Nutrients.

[36] Wang Y., Yang M., Lee S.G., Davis C.G., Koo S.I., Chun O.K. (2012). Dietary total antioxidant capacity is associated with diet and plasma antioxidant status in healthy young adults. J. Acad. Nutr. Diet..

[37] Yang M., Chung S.J., Floegel A., Song W.O., Koo S.I., Chun O.K. (2013). Dietary antioxidant capacity is associated with improved serum antioxidant status and decreased serum C-reactive protein and plasma homocysteine concentrations. Eur. J. Nutr..

[38] Wesson R.N., Sparaco A., Smith M.D. (2008). Chronic pancreatitis in a patient with malnutrition due to anorexia nervosa. JOP.

[39] Theys N., Clippe A., Bouckenooghe T., Reusens B., Remacle C. (2009). Early low protein diet aggravates unbalance between antioxidant enzymes leading to islet dysfunction. PLoS ONE.

[40] Casado A., de la Torre R., López-Fernández E., Carrascosa D., Venarucci D. (1998). Superoxidedismutase and catalaselevels in diseases of theaged. Gac. Med. Mex..

[41] Flores-Mateo G., Carrillo-Santisteve P., Elosua R., Guallar E., Marrugat J., Bleys J., Covas M.I. (2009). Antioxidant enzyme activity and coronary heart disease: Meta-analyses of observational studies. Am. J. Epidemiol..

[42] Jiao Y., Zhang J., Yan J., Stuart J., Gibson G., Lu L., Williams R., Wang Y.J., Gu W. (2011). Differential gene expression between wild-type and Gulo-deficient mice supplied with vitamin C. Genet. Mol. Biol..

[43] Guo C.H., Liu P.J., Hsia S., Chuang C.J., Chen P.C. (2011). Role of certain trace minerals in oxidative stress, inflammation, CD4/CD8 lymphocyte ratios and lung function in asthmatic patients. Ann. Clin. Biochem..

[44] Díaz-Castro J., López-Frías M.R., Campos M.S., López-Frías M., Alférez M.J., Nestares T., Ojeda M.L., López-Aliaga I. (2012). Severe nutricional iron-deficiency anaemia has a negative effect on some bone turnover biomarkers in rats. Eur. J. Nutr..

[45] Sofic E., Rustembegovic A., Kroyer G., Cao G. (2002). Serum antioxidant capacity in neurological, psychiatric, renal diseases and cardiomyopathy. J. Neural. Transm..

[46] Ashour M.N., Salem S.I., el-Gadban H.M., Elwan N.M., Basu T.K. (1999). Antioxidant status in children with protein-energy malnutrition (PEM) living in Cairo, Egypt. Eur. J. Clin. Nutr..

[47] Noor R., Mittal S., Iqbal J. (2002). Superoxide dismutase-applications and relevance to human diseases. Med. Sci. Moni..

[48] Atli T., Keven K., Avci A., Kutlay S., Turkcapar N., Varli M., Aras S., Ertug E., Canbolat O. (2004). Oxidative stress and antioxidant status in elderly diabetes mellitus and glucose intolerance patients. Arch. Gerontol. Geriatr..

[49] Badid N., Ahmed F., Merzouk H., Belbraouet S., Mokhtari N., Merzouk S., Benhabib R., Hamzaoui D., Narce M. (2010). Oxidant/antioxidant status, lipids and hormonal profile in overweight women with breast cancer. Pathol. Oncol. Res..

